# Autoimmunity in Parkinson's Disease: The Role of α-Synuclein-Specific T Cells

**DOI:** 10.3389/fimmu.2019.00303

**Published:** 2019-02-25

**Authors:** Francesca Garretti, Dritan Agalliu, Cecilia S. Lindestam Arlehamn, Alessandro Sette, David Sulzer

**Affiliations:** ^1^Department of Pathology and Cell Biology, Columbia University Irving Medical Center, New York, NY, United States; ^2^Department of Neurology, Columbia University Irving Medical Center, New York, NY, United States; ^3^Division of Vaccine Discovery, La Jolla Institute for Immunology, La Jolla, CA, United States; ^4^Department of Medicine, University of California, San Diego, La Jolla, CA, United States; ^5^Department of Pharmacology, Columbia University Irving Medical Center, New York, NY, United States; ^6^Department of Psychiatry, Columbia University Irving Medical Center, New York, NY, United States

**Keywords:** Parkinson's disease, T cells, adaptive immune system, autoimmunity, α-synuclein, neuroinflammation, blood-brain barrier

## Abstract

Evidence from a variety of studies implicates a role for the adaptive immune system in Parkinson's disease (PD). Similar to multiple sclerosis (MS) patients who display a high number of T cells in the brain attacking oligodendrocytes, PD patients show higher numbers of T cells in the ventral midbrain than healthy, age-matched controls. Mouse models of the disease also show the presence of T cells in the brain. The role of these infiltrating T cells in the propagation of disease is controversial; however, recent studies indicate that they may be autoreactive in nature, recognizing disease-altered self-proteins as foreign antigens. T cells of PD patients can generate an autoimmune response to α-synuclein, a protein that is aggregated in PD. α-Synuclein and other proteins are post-translationally modified in an environment in which protein processing is altered, possibly leading to the generation of neo-epitopes, or self-peptides that have not been identified by the host immune system as non-foreign. Infiltrating T cells may also be responding to such modified proteins. Genome-wide association studies (GWAS) have shown associations of PD with haplotypes of major histocompatibility complex (MHC) class II genes, and a polymorphism in a non-coding region that may increase MHC class II in PD patients. We speculate that the inflammation observed in PD may play both pathogenic and protective roles. Future studies on the adaptive immune system in neurodegenerative disorders may elucidate steps in disease pathogenesis and assist with the development of both biomarkers and treatments.

## Introduction: Parkinson's Disease and Inflammation

Multiple studies have highlighted an association between sustained inflammation and a number of neurodegenerative diseases including Alzheimer's disease (AD), Parkinson's disease (PD), amyotropic lateral sclerosis (ALS), and frontal temporal dementia (FTD) (1). The role of inflammation in the pathogenesis of these disorders, however, remains undetermined. Here, we focus on the inflammatory features in PD, the most common movement disorder, affecting more than 10 million people worldwide (2). PD patients manifest motor symptoms including bradykinesia, rest tremor, muscular rigidity, and postural and gait impairment, as well as non-motor symptoms (3). Non-motor symptoms include mood disorders, cognitive impairments, and autonomic dysfunction, such as orthostatic hypotension and constipation (3). While alleles of many genes are associated with the disorder, PD remains largely (~90% of cases) a sporadic disorder associated with older age and various genetic and environmental risk factors (4).

PD is diagnosed from motor symptoms (3), but non-motor symptoms are often manifest during a prolonged prodromal phase as much as 20 years prior to the onset of the motor features (5). These prodromal non-motor symptoms include constipation, rapid eye movement (REM) sleep behavior disorder, depression, anosmia, and excessive daytime sleepiness (6). The sensitivity and the specificity of these non-motor symptoms limits their utility in predicting the development of PD (6).

Pathologically, PD brains are characterized by progressive degeneration of dopaminergic neurons in the substantia nigra (SN) pars compacta and Lewy pathology. Misfolded α-synuclein (α-syn) becomes insoluble and aggregates to form intracellular inclusions within the neuronal cell bodies (Lewy bodies) and processes (Lewy neurites) (7). Lewy pathology is not only restricted to the central nervous system (CNS) but is also present in the peripheral nervous system (PNS), cardiac plexus, sympathetic ganglia, enteric nervous system (ENS), and other neurons (8). α-Syn aggregates are hypothesized to progress from the periphery, through the spinal cord, into the midbrain and from there across multiple cortical brain regions, as classified in Heiko Braak's staging system (8).

Also noted but often overlooked is the degree of inflammation in PD, both in the periphery and in the brain. Autoreactive T lymphocytes, autoantigen presentation, and microglial activation are detectable in PD patients and animal models of the disease. The recent identification of α-syn-specific T cells in PD patients compounded with α-syn pathology and misprocessing provides a basis for the hypothesis that PD may have features of an autoimmune disorder. It is possible that inflammatory characteristics during and after the prodromal phase and disease might be related. These features, including the adaptive immune response, could play a role in PD pathogenesis or be of diagnostic value even during the prodromal phase.

## Patient Data: Peripheral Pathology, Changes in T Cell Subpopulations, Lymphocyte Infiltration into the CNS and their Roles in PD Etiology

### Peripheral Pathology

PD patients display symptoms of gut dysmotility and altered microbial composition in the intestine, but the cause of these disruptions and their role in pathogenesis remain unclear. Constipation is often an early symptom of PD, preceding motor symptoms by more than 10 years (9, 10) and worsens with disease progression (11). Clinical studies have revealed altered gut microbiota composition in PD patients that may either increase inflammation (12, 13) or gut permeability (14, 15). Indeed, higher intestinal permeability has been observed early in PD pathogenesis (16, 17) and has been associated with enteric α-syn pathology (18). A leaky intestine permits the translocation of the microbes and their products, such as LPS, which initiate sustained inflammation, as well as cells involved in inflammatory responses. It is possible that sustained inflammation can drive α-syn deposition into the ENS. Chen et al. demonstrated that aged Fisher rats fed amyloid-protein curli-producing *Escherichia coli* display increased neuronal expression of α-syn in the submucosal and myenteric plexus of the gut as well as in the brain (19). Possible effects of altered microbiota in PD were illustrated in an α-syn transgenic mouse of PD (20). Transgenic mice grown in germ-free environments exhibited milder symptoms than mice with regular gut microbiota (20). In addition, germ-free mice that were transplanted with PD patient microbiomes displayed worsened motor dysfunction (20).

Influential studies by Braak et al. identified the dorsal motor nucleus of the vagus (DMV) and the ENS of PD patients as early locations for Lewy pathology prior to the *substantia nigra* (8, 21, 22). They hypothesize that α-syn deposition begins in the gut and travels through the vagus nerve into the CNS (8). α-Syn labeling in nerve fibers of the colon is observed in early stage untreated PD patients but is absent in healthy controls or irritable bowel syndrome patients (23), although these findings have not been confirmed in large autopsy cohorts (24, 25).

The chronology of prodromal symptoms has been investigated in a rotenone mouse model of PD. Exposure to rotenone, a pesticide that inhibits complex I of the mitochondrial respiratory chain (26), is linked to PD (27). Chronic, intragastric administration of low doses of rotenone to mice for 1.5 months causes α-syn aggregation in the ENS, DMV, and intermediolateral nucleus of the spinal cord without motor dysfunction (28). Gut motility impairments are observed after 2 months of rotenone treatment (29). After 3 months, α-syn aggregation and loss of dopaminergic neurons is observed in the SN (28). Moreover, α-syn released by enteric neurons may be taken up by presynaptic sympathetic neurites and retrogradely transported to the soma in this model (29). The intragastric rotenone model of PD has been claimed to accurately recapitulate the spatiotemporal development of pathological and clinical symptoms and supports the Braak hypothesis that α-syn pathology begins in the periphery and retrogradely ascends the CNS (8).

Gut pathology is also linked to intestinal inflammation in PD patients. Increased levels of pro-inflammatory cytokines, such as TNFα (tumor necrosis factor α), interleukin (IL)-1β, IL-6, and IFNγ (interferon-γ), are observed and are negatively correlated with disease duration (30). In addition, CD4^+^ T cells infiltrate the colonic mucosa of PD patients with constipation at higher numbers than in PD patients without constipation (31). The gut may be an initiating site of inflammation and pathology and could be the location in which the adaptive immune system is primed against α-syn deposition.

### Changes in T Cell Subpopulations and Cytokines

Consistent with the “systemic” view that PD involves multiple systems and tissues, several studies have shown general alterations in cytokines and immune cell populations.

Proinflammatory cytokines are elevated in the blood of PD patients, including increased levels of IL-2 (32, 33)−6 (34–38)−8 (38), MCP-1 (monocyte chemoattractant protein-1) (38), MIP-1α (macrophage inflammatory protein-1 α) (38), RANTES (regulated upon activation, normal T-cell expressed and secreted) (38, 39), TNFα (35, 36, 40, 41), and IFNγ (38). Increased levels of proinflammatory cytokines and chemokines are indicative of an immune system responding to tissue damage and/or foreign molecules. The levels of cytokines and chemokines correlate with the clinical stage of the disease, highlighting a role for peripheral inflammation in PD progression (38). Altered T cells populations can also contribute to the changes in circulating cytokines. Th1 and TH17^+^ CD4^+^ cells can contribute to the increased levels of IFNγ, TNFα, and IL-17 (42, 43).

Several studies have compared the T cell subpopulations in PD patients with those of healthy controls. PD patients are found to have fewer naïve T cells (44, 45), including CD4^+^ Th (helper) cells (44, 46, 47), and more activated T cells (48), including Th2 regulatory T cells (Tregs) (44, 46), than healthy age-matched controls. Some studies have reported a general decrease in total Th cells (46, 49, 50), Tregs (42, 51), and CD8^+^ cytotoxic T cells (50), whereas other studies show no change in Treg number (52) with an increase in Th1 cells, but more significantly the Th17 subset in PD patients (43, 51, 53). These disparate findings in the levels of naïve, helper, cytotoxic, and regulatory T cells in PD may be due to differences in phenotyping methods and patient recruitment. Note further that these studies only examine the total bulk populations of T cells without discerning changes potentially related to autoantigen recognition, which may represent a very small fraction of the total populations. It will be important to reexamine these results in light of the recent growing definition of the antigen specificities of T cells associated with neurodegenerative diseases.

The number and proportion of T cells types provide clues to their roles. Tregs respond to heightened inflammation by suppressing effector T cell proliferation and cytokine production (54) and their dysregulation can lead to autoimmune disorders. PD patient-derived Tregs have been shown to have an impaired ability in suppressing effector T cell proliferation *in vitro* compared to Tregs of healthy controls (45). In addition, effector T cells from PD patients produce higher amounts of proinflammatory cytokines in response to T cell activators *in vitro* and their Tregs fail to suppress cytokine release (42). If Tregs are dysfunctional in PD, the heightened and persistent proinflammatory environment observed in patients may remain unabated throughout the nervous system and periphery.

### Blood-Brain Barrier Breakdown

Increased levels of peripheral cytokines act on CNS endothelial cells that form the blood-brain barrier (BBB) to increase vascular permeability (55). The CNS was formerly considered an immunologically privileged site, largely due to the presence of the BBB, a physical barrier formed by endothelial cells via interactions with pericytes and astrocytes to prevent blood proteins, antibodies, immune cells, and drugs from penetrating into the brain parenchyma (56). The BBB, however, becomes disrupted during acute and chronic inflammation. Under sustained chronic inflammation, tight junctions between endothelial cells that prevent paracellular diffusion are weakened or disrupted, allowing the passage of antibodies or immune cells that would otherwise be restricted from the brain (57). In addition, inflamed CNS endothelial cells upregulate expression of adhesion molecules (e.g., ICAM and VCAM-1) that bind and recruit circulating T cells and monocytes, as well as proteins (e.g., caveolin-1, PLVAP) that promote transcellular transport of immune cells and antibodies across the barrier (55, 58). A compromised BBB renders the brain susceptible to circulating immune cells, antibodies, and pro-inflammatory cytokines. T cells can then extravasate into the brain via the disrupted BBB, as seen in both MS patients and animal models for the disease (55, 58).

Neuroimaging studies on PD patients show that the BBB is compromised in the vicinity of the midbrain (59) as well as various deep and cortical gray matter and white matter regions (60, 61). In post-mortem tissue, there is evidence of brain capillary leakage indicated by deposition of blood-derived proteins (e.g., fibrinogen and IgG) in the striatum and subthalamic nucleus (62, 63). Capillary leakage associated with microvascular degeneration, disrupted tight junctions, changes in capillary basement membrane composition in the subthalamic nucleus (63), and aberrant angiogenesis (marked by increased levels of integrin αvβ3, a proangiogenic integrin expressed by immature endothelial cells) have been found within the SN, locus coeruleus, and putamen of PD brains (64).

## Lymphocyte Infiltration and its Role in PD Etiology

### T Cells in the Midbrain of PD Patients

Perhaps as a result of peripheral inflammation, changes in lymphocyte subtype populations, and BBB breakdown, T cells can infiltrate the affected brain regions of PD patients. First reported by the McGeers in 1988, CD3^+^ cells, a marker for T cells, were detected within the CNS of PD brains (65, 66). Brochard et al. showed that both CD4^+^ and CD8^+^ T cells, but not B and natural killer (NK) cells, infiltrated the SN of PD patients and were present at much far greater levels than in healthy controls. These T cells were located near blood vessels and neuromelanin-containing dopaminergic neurons (43, 67). The presence of T cells in the region affected in the disease suggests a targeted extravasation, rather than a random consequence of increased BBB permeability by peripheral inflammation. If T cells that had escaped self-tolerance circulate in the blood of PD patients, it is plausible that they could infiltrate into the brain permitted by a leaky BBB. The causal role of infiltrating T cells is further elucidated in studies from mouse models of PD.

### Data From Animal Models Support a Role for T Cells in Disease Pathogenesis

Although animal models have limitations in recapitulating PD, they are useful tools for genetic manipulation and identifying features of disease pathology. A range of models for studying PD have been developed, employing toxins or genetic mutations that recapitulate certain aspects of the disease. Most of the models highlight a causative role for infiltrating T cells in propagating neurodegeneration.

#### Toxin Models of PD

Intracerebral injections of the toxin 6-hydroxy-dopamine (6-OH-DA) into the midbrain of mice induces degeneration of dopamine and noradrenaline neurons (68), which are rendered vulnerable to the drug because they express the dopamine transporter, DAT, that accumulates the toxin. Once in the neuronal cytosol, 6-OHDA mediates oxidative stress-related cytotoxicity (69–71). The subsequent acute neurodegeneration is manifested in motor deficits in mice and, if injected unilaterally, in rotational motions. 6-OHDA treated mice show IgG leakage, indicative of a leaky BBB, as well as T and B cell infiltration around CNS blood vessels (72). Treg cells are significantly decreased in the periphery of 6-OHDA treated rats, which are reported to not show marked T cell infiltration into the midbrain (73).

Another toxin model uses 1-methyl-4-phenyl-1,2,5,6-tetrahydropyridine (MPTP), originally identified as a contaminant in intravenous drug users who developed PD (74, 75). MPTP is lipophilic and readily crosses the BBB and it is then internalized by astrocytes where it is metabolized to MPP^+^. Astrocytes release MPP^+^, which is taken up by dopaminergic neurons via DAT (76, 77). Once accumulated, MPP^+^ induces SN death by inhibiting complex I in the electron transport chain, depleting cells of ATP, and increasing oxidative stress in a dopamine and calcium-dependent manner (78–81).

T cells extravasate into the SN as soon as 2 days post MPTP treatment (82, 83), following microgliosis (67). A causative role for T cells in MPTP-induced dopaminergic degeneration was investigated using immunodeficient mouse strains. Mice that lack functioning T cells (*Rag1*^−/−^ and *Tcrb*^−/−^ mice) are protected from MPTP-induced neurodegeneration. Despite the observation that CD8^+^ T cells are more numerous in the midbrain following MPTP, *Cd8a*^−/−^ mice were not protected from neuronal death. Mice that lack functioning CD4^+^ (*Cd4*^−/−^ mice) cells, however, were protected from loss of dopaminergic neurons (67), implicating this arm of the adaptive immune system. SCID mice, which are deficient in functional T and B cells, were also protected against MPTP induced neuronal death (84). Neurodegeneration was restored in SCID mice that are reconstituted with wild-type splenocytes (84). These studies reinforce the notion that a functioning immune system, and CD4^+^ T cells in particular, may be essential for neurodegeneration in the MPTP model of PD.

Interestingly, dopamine (DA) receptors are expressed by cells of the innate and adaptive immune response, including CD8^+^ and CD4^+^ T cells. Expression of the D3-type receptor (D3R), one of the five DA receptors in humans, was significantly reduced in PD patient T cells and is correlated with disease severity (85). D3R-deficient mice treated with MPTP were protected from dopaminergic neuron death and microglial activation, but the protection was lost with the transfer of CD4^+^ T cells from wild-type animals (86). Additionally, *Rag1*^−/^^−^ mice that were protected against MPTP become susceptible to neurodegeneration with the transfer of WT CD4^+^ T cells but not when reconstituted with D3R-deficient CD4^+^ T cells (86). These data suggest that D3R signaling in T cells may be necessary in the MPTP mouse model.

T cell receptor stimulation is reported to induce D3R expression on CD4^+^ T cells, and D3R signaling on CD4^+^ T cells contributes to the expansion of Th1 and Th17 cells in the context of inflammatory colitis (87). DA is in part depleted in PD, as a subset of dopaminergic and norepinephrinergic neurons are lost in the disease. The role of DA receptors on T cells could evolve over the disease course, including via decreased activation due to DA loss and spikes of activation with treatment of DA replacing drugs. The level and activation profile of these receptors throughout the disease could shed light on their role in contributing to a proinflammatory T cell profile, particularly during drug treatment.

While CD8^+^ and CD4^+^ Th1 T cells can exert cytotoxic and proinflammatory effects, CD4^+^ Tregs exert anti-inflammatory properties that protect cells against neurotoxic molecules or persistent inflammation. Adoptive transfer of Th cells, induced by co-polymer immunization, into MPTP mice protects dopaminergic neurons. This protection seems to be mediated via suppression of microglial activation (88–91).

#### Viral Overexpression of α-Syn to Model PD

A different approach to modeling PD uses targeted overexpression of human α-syn in the midbrain of rodents. In one model, α-syn is overexpressed in the SN by injection with recombinant adeno-associated virus vector (AAV2-SYN), which leads to the slow degeneration of dopaminergic neurons (92). Consistent with findings from the toxin mouse models above, AAV2-SYN injected mice display IgG deposition as a consequence of BBB breakdown, as well as T and B cell infiltration (92). The AAV2-SYN model induces a slower, less acute neurodegeneration than the toxin-based PD approaches, and so may prove useful for understanding the timing of inflammation and neuronal death. Importantly, human α-syn overexpression triggers microglial activation, BBB leakage, and recruitment of T and B cells into the SN *prior* to neurodegeneration in mice (92). These findings support the concept that immune cell recruitment and local inflammatory features play roles in neurodegeneration, rather than that they are consequences downstream from neuronal death.

In a rat model of PD, overexpression of a rare, autosomal dominant A53T mutant α-syn allele that causes α-syn aggregation and early onset PD in humans, induces microgliosis and lymphocytic infiltration in SN (93). However, administration of FK506, an immunosuppressive drug that inhibits T cell signal transduction and IL-2 transcription, on the rAAv2/7 α-syn overexpressing rat model increased the survival of dopaminergic neurons, with a positive trend for motor improvement. FK506 reduced the numbers of microglia, macrophages, and CD4^+^ T cells in the SN and delayed the infiltration of CD8^+^ T cells (93).

### *In vitro* Studies of iPSC-Derived Dopamine Neurons With Th17 Cells

A recent *in vitro* study using induced-pluripotent stem cells (iPSC)-derived midbrain neurons and T cells from PD patients was the first to show that PD patient-derived T cells can kill dopamine neurons directly. Sommer et al. determined that PD patients contain significantly higher Th17 cells than healthy controls (43). The PD patient-derived Th17 cells exerted cytotoxic effects on neurons by releasing IL-17A, a cytokine detected by IL-17R expressed on neurons (43). The iPSC *in vitro* cultures lacked glia, which express MHC-II and can potentially interact with Th17 cells. In addition, T cells were activated non-specifically, and so the antigen specificity of Th17 cells remains unclear (43). While the study indicates that PD-derived T cells can directly kill dopaminergic neurons, the omission of professional antigen presenting cells, antigenicity, and neuronal specificity in the cultures in this initial study overlooks the role of multiple relevant *in vivo* factors important for disease progression. Moreover, the mode of action that garners specific vulnerability of dopaminergic neurons and avoids unaffected neurons was not resolved in this study. Nevertheless, the study indicates Th17 cells may participate in PD-related neuronal death.

Th17 cells play a prominent role in multiple sclerosis (MS), a well-characterized autoimmune neurological disease. MS patients suffer from white and gray matter lesions of neuronal demyelination and axonal loss due to a dynamic interplay between the adaptive immune system, glia, and neurons (94). The origin of the primary lesion and subsequent inflammation is heavily debated; however, studies in patients and animal models highlight a critical role for T and B cells in MS pathogenesis (95). Increased serum levels of IL-17A and Th17 cells in MS patients has been reported, ranging from 0.77 to 2.48 and 0.34 to 1.55% of total CD4^+^ T cells in MS patients compared to healthy controls, respectively (96). These levels are higher than those detected in PD patients (43). In the experimental autoimmune encephalomyelitis (EAE) model of MS, myelin oligodendrocyte glycoprotein-specific Th17 cells form direct contacts with neuronal cells in demyelinating lesions and are associated with extensive axonal damage (97). Th17 cells seem to exert their neurotoxic effects by releasing IL-17A, which interferes with the remyelination process (98) and directly damages the BBB (99). Th17 cells also release GM-CSF, which is essential for inducing EAE (100, 101) and likely supports the recruitment of peripheral monocytes (100, 102), and IFNγ that activate infiltrating macrophages (103). Interestingly, studies on the mode of action of Th17 cells in EAE do not include IL17R mediated neurotoxicity. Studies on the role of Th17 in MS patients and mouse models (EAE), could be emulated in PD mouse models to further determine the role and mode of action of Th17 lymphocytes in disease pathogenesis.

### The Role of MHC Proteins in PD Pathogenesis

T cells recognize a complex formed between MHC molecules (known as HLA; human leukocyte antigen in humans) and a peptide epitope. The epitope is presented to T cells as a result of a complex series of events termed antigen processing and presentation that involve the intracellular degradation of self and foreign proteins into peptides that are then loaded onto the antigen-binding groove of MHC molecules. In general, T cells recognize peptides derived from foreign molecules, but in disease some T cells also evade self-tolerance and can recognize self-peptides. MHC class I in general presents peptides derived from the same cell (endogenous presentation), while MHC class II typically present peptides derived from extracellular proteins (exogenous presentation).

Mature neurons are generally thought to lack MHC expression and so to not present antigens. However, SN and LC neurons recently have been shown to express MHC-I and co-localize with CD8^+^ T cells in response to stimuli such as IFNγ (104). Catecholamine neurons are particularly sensitive to inflammation in comparison to other neuron types. A lower amount of IFNγ is required by dopaminergic neurons to express MHC-I in comparison to other neuronal subtypes. The MHC-I presented by dopaminergic neurons is functional and can result in cytotoxicity in the presence of matching antigen and CD8^+^ T cells (104). Dopaminergic neuron sensitivity to inflammation and corresponding MHC-I upregulation could render them susceptible to infiltrating T cells versus neighboring, more resistant GABAergic neurons.

GWAS in PD have shown an association with haplotypes of MHC II genes, including DRB5^*^01 and DRB1^*^15:01 alleles and a polymorphism in a non-coding region that may increase levels of MHC class II expression (105–109). MHC-II positive microglia are detected in the SN of PD patients (65) and their levels increase with disease severity (110).

Animal models of PD have been employed to investigate the role of MHC in neurodegeneration. AAV2- α-syn overexpression in mice that lack MHC-II are protected against microglial activation, BBB permeability, and neuronal death in the SN (111). A necessity for MHC-II signaling for riving neuronal death is reported in the MPTP model of PD. MHC-II null mice are resistant to neuronal death by MPTP intoxication and their microglia do not release pro-inflammatory cytokines in contrast to wild-type animals (112).

Association of HLA alleles to late-onset PD and a positive correlation between levels of MHC-II expression and disease severity in humans, in conjunction with the necessity for MHC-II expression in PD mouse models, suggests that antigen presentation and adaptive immune signaling may be involved in dopaminergic neuron death. Infiltrating T cells, MHC expression, and sensitivity of dopaminergic neurons to inflammation support the concept that PD possesses features of an autoimmune disorder.

### The Connection With Innate Immunity and Antigen Presenting Cells

While functioning T cells and MHC signaling may be critical in modeling PD pathology, activated peripheral antigen presenting cells and microglia are potential initiators and drivers of neuroinflammation. Professional antigen presenting cells detect foreign pathogens via pattern recognition receptors (PRR), such as toll-like receptors (TLRs), activating signaling cascades that change the inflammatory profile of the cell (113). Picomolar concentrations of α-syn, possibly relevant to extracellular levels in PD, sensitized the response by macrophages to release proinflammatory cytokines (114). Extracellular neuromelanin derived from human autopsy also activates microglia via CD11b/ CD18 (also known as Mac-1) integrin receptors (115). Thus, it is not surprising that peripheral blood monocytes isolated from PD patients have been shown to be hypersensitive and predisposed to an inflammatory stimulus (116). Antigen presenting cells in the periphery have the potential to be the first in line to respond to sustained, slight increases in α-syn levels, which have deleterious effects over time. In addition, *in vitro* studies have shown that α-syn acts as a chemoattractant to neutrophils and monocytes (117). Peripherally activated antigen presenting cells such as monocytes can extravasate into different tissues, including the brain, during active disease states (118). In an AAV overexpressing model of PD, α-syn expression recruited peripheral monocytes to the brain and their recruitment was necessary for dopaminergic neuron death (119). α-Syn in the periphery and CNS acts as an activator and recruiter of professional antigen presenting cells, potentially initiating an inflammatory response against itself.

Microglia are the primary immune cells of the CNS and act as both resident phagocytic and antigen presenting cell to provide an active immune defense (120). Microgliosis is characterized by microglia proliferation, change in morphological state from ramified to amoebic, and the presence of several inflammatory markers, such as CD68 (120). Microglial activation is an important potential player in PD-associated inflammation and immune reactivity. Neurons do not express Class II, while microglia are MHC Class II expressing antigen-presenting cells. MHC Class II present peptides derived from extracellular proteins (exogenous presentation), and cells of the monocyte lineage such as microglia are particularly apt at internalizing aggregated proteins (e.g., α-syn aggregates) and presenting peptides derived from exogenous proteins in the context of class II MHC molecules. They therefore represent a strong candidate for MHC class II restricted presentation of α-syn peptides to T cells.

*In vivo* imaging studies of microglial activation using positron-emission tomography (PET) showed that PD patients have markedly increased neuroinflammation in various brain regions, including the basal ganglia and striatum, regardless of the number of years with disease (121, 122). Studies have determined that inhibiting or preventing microglial activation renders PD mouse models resistant to neuronal death. RANTES and eotaxin, two chemokines secreted by activated microglia, are upregulated in MPTP mice and monkeys (123, 124). Treatment of MPTP monkeys with NEMO-binding domain (NBD) peptide, which selectively inhibits NF-κB activation, decreases microglia expression of RANTES and attenuates CD8^+^ T cell infiltration into the SN (123). Neutralization of RANTES and eotaxin in MPTP-intoxicated mice decreases lymphocyte infiltration in the SN, reduces inflammation, and confers dopaminergic neuronal protection and improved motor function (124). The neuron-derived chemokine fractalkine (CX3CL1) is a ligand for CX3CR1 on microglia and promotes sustained microglial activation. Mice that are CX3CR1-deficient are protected against dopaminergic neuron loss in MPTP-treated mice (125). CX3CR1 in microglia are likely limiting the neurotoxic effect of CCL2 induction by astrocytes, which promotes CCR2^+^ monocyte infiltration (126). Treatment of 6-OHDA rats with safinamide suppressed microglial activation and protected dopaminergic neurons from degeneration (127). In addition, deficits in microglial restraint exacerbate dopaminergic neurodegeneration in 6-OHDA rats (128). Taken together, these studies show that microglial activation is critical for immune cell recruitment and neuronal death in PD. α-Syn itself may activate microglia and antigen presenting cells, initiating an immune response in the brain and periphery. Peripheral α-syn-specific T cells may migrate to the brain and be further activated by α-syn-presenting microglia that have internalized aggregates.

### A Model of α-Syn Recognition in PD

It is possible that the innate immune system in the SN is activated and processes and presents α-syn aggregates to specific T cells in the brain region prior to neuronal death. While it is unclear which factors activate microglia, numerous *in vitro* studies show that α-syn or conditioned media from α-syn expressing neurons robustly activate microglia. Treatment of primary microglia with recombinant, monomeric α-syn consistently induces an activated, pro-inflammatory profile, such as increased TNFα, IL-1β, IL-6, COX-2 expression, and iNOS production. When α-syn is overexpressed in mouse models, either via virus injection or transgenically, it initiates an immune response via upregulation of microgliosis and recruits lymphocytes into the SN, prior to degeneration [for review (129)].

Autoimmunity arises from the failure to recognize endogenous proteins as self, driving the immune system to respond to and attack ones own cells and tissues. PD might result from an autoimmune response by recognizing aggregated, misprocessed α-syn as a foreign entity. α-Syn is both hyperphosphorylated and proteolytically misprocessed in the brains of PD patients, leading to its aggregation and deposition (130). Modified forms of α-syn are resistant to normal degradation by the proteasome and chaperone-mediate autophagy (CMA) (131). Proteolytic misprocessing of mutant or aggregated protein or post-translation modifications of α-syn in PD (131) could potentiate the formation of neo-epitopes.

Peptides derived from α-syn elicit *in vitro* responses from cytotoxic and Th cells from PD patients but not healthy controls (132). In addition, one of these peptide regions strongly binds to MHC complexes encoded by HLA (DRB1^*^15:01, DRB5^*^01:01) that are associated with PD by GWAS (105–109). The second region of these peptides requires phosphorlyaton at an α-syn residue, S129, that is found in high levels in Lewy bodies (133). We note that these α-syn-specific T cells have thus far only been detected in the periphery of PD patients. If they were to extravasate into the brain, they would detect α-syn on the surface of microglia and neurons. Antigen recognition by Th1 and cytotoxic T cells leads to increased local inflammation and cell death. However, whether α-syn–specific T cells identified in PD patients permeate into the CNS and cause dopaminergic neuron death remains to be determined.

### Implications for Diagnosis and Therapeutics

Understanding the extent of the role of the immune response in the pathogenesis of PD opens up new avenues of diagnosis and treatment for patients. Identifying people who are carriers of HLA alleles that place them at risk for α-syn-specific T cells to be monitored can help early diagnosis and treatment of PD. In addition, α-syn-specific T cells could be use as early biomarkers of the disease, identifying autoimmunity to self proteins prior to the onset of motor symptoms. Large-scale, longitudinal studies monitoring α-syn reactivity and the development of PD would need to be conducted before establishing T cells as PD biomarkers.

Currently, new treatment avenues targeting the immune system are being tested on PD patients. Sargramostim is a human recombinant granulocyte-macrophage colony-stimulating factor approved by the Food and Drug Administration for the recovery of patients receiving bone marrow transplantation and cancer therapies (134). It functions by promoting myeloid recovery and inducing Treg responses (134). In a preliminary randomized, double-blind phase 1 clinical trial, Sargramostim-treated patients showed modest improvements after 6 and 8 weeks of treatment and increases in Treg numbers and function compared to PD patients receiving placebo (135). Further clinical investigation will shed light on the potential of immunomodulatory drugs for treatment of PD.

Another approach to treatment currently being investigated is the targeting of α-syn with antibodies to slow the spread and reverse the effects of α-syn pathology. Monoclonal antibodies against α-syn have shown to reduce the levels of protein spread, ameliorate dopaminergic neuron loss, and attenuate motor deficits in PD mouse models (136, 137). Affitope PD01A and PRX002/RG7935 currently in clinical trial have shown to penetrate the BBB and lower plasma levels of α-syn (138, 139). However, it should be noted that anti-α-syn antibodies are shown to be present in the blood and cerebral spinal fluid of PD patients and absent in healthy controls (140–142). Therefore, anti-α-syn antibodies may be more promising as an early biomarker for PD rather than a treatment.

The immune system is increasingly acknowledged to play a larger role in propagating PD than previously thought and is a promising pathway for treatment. When designing immune-modulatory drugs, timing is important. If peripheral inflammation, α-syn aggregation, BBB leakage, and α-syn-specific T cells drive neuronal death, it is critical to treat PD patients before the activation of the autoimmune pathway. Immunomodulatory treatment would be most efficacious at the onset of loss of α-syn tolerance, before immune cells have targeted dopaminergic cells. Developing biomarker tests to examine patients and identify α-syn-specific T cells early would provide a longer window for immunomodulatory treatments to stop PD progression in its tracks.

## Conclusions and Future Directions

**Figure 1:**
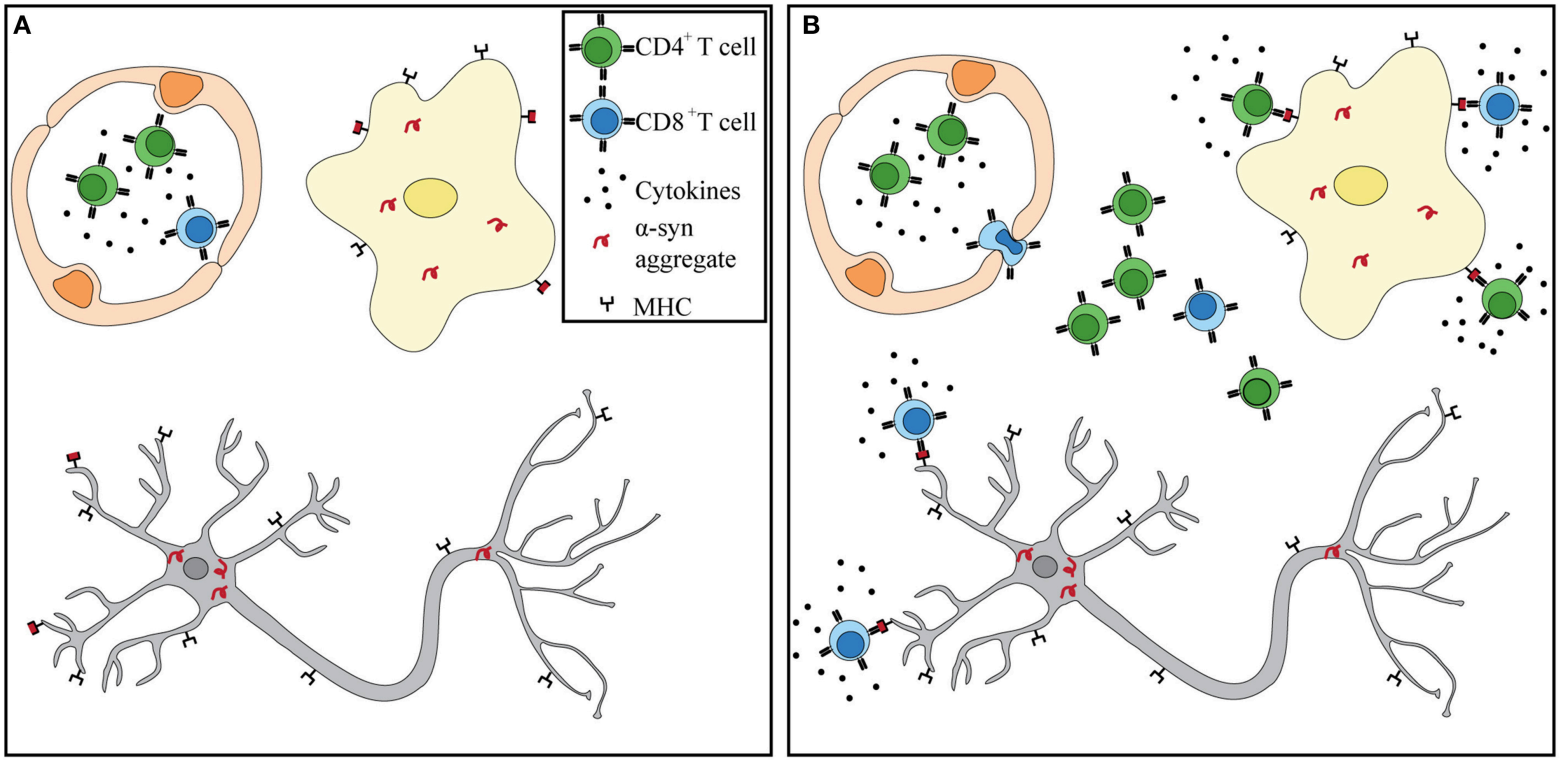
α-Syn T cells infiltrate into the CNS and recognize antigen presented by microglia and dopaminergic neurons. **(A)** Prior to BBB damage, peripherally primed α-syn T cells circulate throughout the blood in the presence of proinflammatory cytokines. α-Syn aggregates have already formed in dopaminergic neurons and microglia. **(B)** Increased BBB permeability through either weakened tight junctions or increased transcytosis allows for extravasation of α-syn T cells. Recognition of α-syn presented by MHC-I on neurons and MHC-I and –II on microglia leads to T cell activation and release of granzymes and proinflammatory cytokines. Dopaminergic neurons eventually die in the presence of sustained inflammation and cytotoxic environment.

Numerous independent studies suggest that a heightened and sustained immune response observed in PD patients may be a driver, rather than a consequence, of neuronal death. α-Syn pathology may begin in the periphery, where the enteric nervous system is compromised up to 20 years prior to diagnosis. Circulating T cells of PD patients have been shown to respond to α-syn stimulation *in vitro*. PD patients are afflicted with sustained peripheral inflammation, which has been shown to lead to a deterioration of the BBB. Lymphocytes can extravasate into the CNS via the BBB that is shown to be leaky in PD patients. Within the CNS, α-syn accumulation and spread, microgliosis, and antigen presentation can help recruit and attract α-syn -specific T cells. α-Syn -targeting CD4^+^ and CD8^+^ T cells observed in PD patients can recognize specific peptides bound to MHC-II on microglia and MHC-I on dopaminergic neurons (Figure 1). Further studies are necessary to test this hypothesis. However, that each phenomenon has been observed in PD patients and proven essential in recapitulating the disease in animal models lends support to its potential. Future studies should integrate α-syn-specificity and HLA association observed in PD patients with iPSCs neurons and antigen presenting cells to fully recapitulate what occurs in the CNS during disease. If α-syn-specific T cells are driving neurodegeneration in PD, then biomarkers, diagnostic tools, and treatments can be adapted to identifying autoimmune cells in patients and targeting them.

## Author Contributions

FG wrote the review. DA, CL, AS, and DS edited the review and provided feedback.

### Conflict of Interest Statement

The authors declare that the research was conducted in the absence of any commercial or financial relationships that could be construed as a potential conflict of interest.
